# Supplementary material for: Glutathione binding to the plant AtAtm3 transporter and implications for the conformational coupling of ABC transporters
Source: eLife. 2022 Mar 25;11:e76140. doi: 10.7554/eLife.76140 (PMC9000953; doi:10.7554/eLife.76140)
Supplement: Supplementary file 1. [file elife-76140-supp1.docx]

|  | **Inward** | **Inward + GSSG** | **Closed** | **Outward** |
| --- | --- | --- | --- | --- |
| **Data Collection and processing** | | | | |
| **Microscope** | Titan Krios at Caltech Cryo-EM facility | Titan Krios at Caltech Cryo-EM facility | Titan Krios at Stanford-SLAC CryoEM Center | Titan Krios at Caltech Cryo-EM facility |
| **Camera** | Gatan K3 | Gatan K3 | Falcon IV | Gatan K3 |
| **Magnification** | x105,000 | x105,000 | - | x105,000 |
| **Voltage (keV)** | 300 | 300 | 300 | 300 |
| **Exposure (e/Å^2^)** | 60 | 60 | 48 | 60 |
| **Pixel size (Å)** | 0.855 | 0.855 | 0.82 | 0.855 |
| **Defocus Range (um)** | - 1.0 to -3.0 | - 1.0 to -3.0 | - 1.5 to -2.1 | - 1.0 to -3.0 |
| **Initial Particle Image (no.)** | 4,608,600 | 2,510,131 | 4,230,175 | 1,675,302 |
| **Final Particle Image (no.)** | 157,762 | 259,020 | 140,569 | 103,161 |
| **Symmetry Imposed** | C2 | C2 | C2 | C2 |
| **Map Resolution (Å)** | 3.4 | 3.6 | 4.0 | 3.8 |
| **FSC Threshold** | 0.143 | 0.143 | 0.143 | 0.143 |
| **Map Resolution Range (Å)** | 3.2 - 4.1 | 3.5 - 4.0 | 3.9 - 4.2 | 3.9 - 4.3 |
| **Refinement** | | | | |
| **Initial Model Used** | PDB ID: 6pam | PDB ID: 6pam | PDB ID: 6par | PDB ID: 6par |
| **Model Resolution (Å)** | 3.4 | 3.57 | 3.95 | 3.81 |
| **FSC Threshold** | 0.143 | 0.143 | 0.143 | 0.143 |
| **Model composition** |  |  |  |  |
| **non-hydrogen atoms** | 9326 | 9238 | 9254 | 9122 |
| **protein residues** | 1200 | 1180 | 1178 | 1160 |
| **ligands** | - | GDS:1 | ADP: 2; MG: 2; VO_4_:2 | ADP: 2; MG: 2; VO_4_:2 |
| **Average B-factors (Å^2^)** |  | | | |
| **protein** | 99.8 | 84.2 | 118.7 | 49.3 |
| **ligands** | - | 60.7 | 62.1 | 25.3 |
| **R.m.s. deviations** |  | | | |
| **Bond length (Å)** | 0.004 | 0.002 | 0.003 | 0.002 |
| **Bond angles (º)** | 0.841 | 0.562 | 0.613 | 0.482 |
| **Validation** |  | | | |
| **MolProbity score** | 1.82 | 1.36 | 1.60 | 1.41 |
| **Clashscore** | 14.6 | 6.6 | 11.8 | 6.3 |
| **Rotamer outliers** | 0 | 0.2 | 0.2 | 0.2 |
| **Ramachandran plot** |  | | | |
| **Ramachandran favored (%)** | 97.2 | 98.4 | 98.0 | 97.7 |
| **Ramachandran allowed (%)** | 2.8 | 1.6 | 2.1 | 2.3 |
| **Ramachandran outliers (%)** | 0 | 0 | 0 | 0 |
| **PDB ID** | 7n58 | 7n59 | 7n5a | 7n5b |
