# Supplementary material for: Glutathione binding to the plant AtAtm3 transporter and implications for the conformational coupling of ABC transporters
Source: eLife. 2022 Mar 25;11:e76140. doi: 10.7554/eLife.76140 (PMC9000953; doi:10.7554/eLife.76140)
Supplement: Supplementary file 2. — Calculated component 1 values in PCA are listed for different transporters. For heterologous transporters, transporter encoded in one polypeptide and transporters with different conformational states in one PDB file, ‘_A/B’ is added at the end of each PDB to represent different half-transporters and/or different conformations. Residues used for distance measurements and the corresponding distance for each transporter are listed. Grayed out cells represent distances that cannot be measured for the second half-transporters of heterodimeric transporters. [file elife-76140-supp2.docx]

| **PDB ID** | **PCA Component 1 (Å^2^)** | **Distances** | | | | | | | |
| --- | --- | --- | --- | --- | --- | --- | --- | --- | --- |
|  |  | **Outside Residues** | **Outside Distances (Å)** | **Coupling helices 1 (CH1) Residues** | **CH1 Distances (Å)** | **Coupling helices 2 (CH2) Residues** | **CH2 Distances (Å)** | **Conserved Glu Residues** | **Glu Distances (Å)** |
| 2hyd | 0 | 278 | 24.6 | 114 | 28.4 | 209 | 14.8 | 503 | 14.3 |
| 3b5w | -252.01 | 282 | 6.4 | 118 | 50.4 | 214 | 70.3 | 506 | 64.3 |
| 3b5x | -95.19 | 282 | 19.9 | 118 | 17 | 214 | 32.6 | 506 | 34.8 |
| 3b5y | -0.09 | 282 | 28.9 | 118 | 29.4 | 214 | 22.7 | 506 | 14.8 |
| 3b5z | 2.61 | 282 | 30.5 | 118 | 30.8 | 214 | 24.4 | 506 | 14.2 |
| 3b60 | -0.59 | 282 | 31.4 | 118 | 29.1 | 214 | 22.6 | 506 | 14.7 |
| 3g5u_A | -112.24 | 325&967 | 15.1 | 161&803 | 28.5 | 257&900 | 37.7 | 552&1197 | 31.4 |
| 3g5u_B | -52.24 |  |  |  |  |  |  |  |  |
| 3qf4_A | -115.57 | 270&297 | 9.8 | 106&130 | 31.7 | 201&227 | 32.4 | 495&517 | 19.3 |
| 3qf4_B | -109.08 |  |  |  |  |  |  |  |  |
| 3wme | -90.79 | 376 | 11.5 | 214 | 23.6 | 309 | 35.3 | 610 | 33.9 |
| 3zdq | -125.91 | 428 | 12.8 | 265 | 31.2 | 361 | 33.6 | 659 | 23.9 |
| 4ayt | -117.99 | 428 | 12.3 | 265 | 26.6 | 361 | 30.6 | 659 | 22.9 |
| 4ayw | -121.61 | 428 | 7.8 | 265 | 24.4 | 361 | 32.8 | 659 | 28.8 |
| 4ayx | -133.59 | 428 | 11.3 | 265 | 31.9 | 361 | 35.6 | 659 | 26.4 |
| 4f4c | -168.99 | 352&1014 | 15.7 | 189&847 | 45.6 | 285&945 | 51.5 | 580&1243 | 48.6 |
| 4mrs | -47.43 | 298 | 10.2 | 134 | 23.8 | 231 | 35.9 | 523 | 31.3 |
| 4myc | -111.84 | 372 | 9.4 | 208 | 39.4 | 305 | 40.9 | 598 | 37.3 |
| 4pl0 | -21.98 | 284 | 15.9 | 119 | 26 | 216 | 22.4 | 506 | 13.2 |
| 4q4a_A | -101.87 | 269&293 | 9.2 | 106&130 | 36.6 | 202&226 | 34.8 | 495&517 | 19.8 |
| 4q4a_B | -110.01 |  |  |  |  |  |  |  |  |
| 4ry2 | -68.98 | 423 | 11.5 | 260 | 34.8 | 335 | 29.6 | 648 | 16.3 |
| 5c73 | -19.25 | 290 | 14.5 | 127 | 25.9 | 222 | 20.6 | 510 | 14.4 |
| 5c76 | -187.37 | 290 | 7.1 | 127 | 48.1 | 222 | 54.9 | 510 | 44.7 |
| 5c78 | -215.98 | 290 | 7.7 | 127 | 51.8 | 222 | 62.7 | 510 | 58.1 |
| 5eg1 | -20.71 | 284 | 15.9 | 119 | 26.3 | 216 | 22.3 | 506 | 13.2 |
| 5koy_A | -122.35 | 324&967 | 12.6 | 161&802 | 36.3 | 257&900 | 38.1 | 552&1197 | 29.7 |
| 5koy_B | -67.46 |  |  |  |  |  |  |  |  |
| 5mkk_A | -97.26 | 275&290 | 9.6 | 112&127 | 23.2 | 208&223 | 32.3 | 500&523 | 25.8 |
| 5mkk_B | -99 |  |  |  |  |  |  |  |  |
| 5nbd | -122.85 | 286 | 7.9 | 127 | 29.9 | 222 | 35.7 | 510 | 18.9 |
| 5ofp | -11.32 | 284 | 16.3 | 119 | 24.8 | 215 | 16.5 | 506 | 17.4 |
| 5ofr | -20.86 | 284 | 16 | 119 | 26.2 | 215 | 16.6 | 506 | 13.1 |
| 5ttp | -22.3 | 283 | 14.1 | 118 | 27.6 | 215 | 22.3 | 506 | 13.4 |
| 5tv4 | -99.97 | 282 | 10.7 | 118 | 26.9 | 215 | 35.1 | 506 | 26.1 |
| 5u1d_A | -129.2 | 407&442 | 15.6 | 243&278 | 25.6 | 339&375 | 39.4 | 632&667 | 41.1 |
| 5u1d_B | -93.49 |  |  |  |  |  |  |  |  |
| 5uak_A | -90.58 | 334&1127 | 8.7 | 174&966 | 38.4 | 270&1061 | 37.6 | 572&1371 | 35.7 |
| 5uak_B | -83.07 |  |  |  |  |  |  |  |  |
| 5uar_A | -86.26 | 335&1135 | 10.8 | 175&974 | 41 | 269&1069 | 32.6 | 571&1292 | 33.5 |
| 5uar_B | -69.55 |  |  |  |  |  |  |  |  |
| 5uj9_A | -137.22 | 579&1226 | 5.2 | 419&1065 | 48.1 | 513&1160 | 45.1 | 792&1454 | 46.8 |
| 5uj9_B | -93.27 |  |  |  |  |  |  |  |  |
| 5uja_A | -87.87 | 579&1227 | 5.1 | 417&1065 | 40.9 | 513&1160 | 35.1 | 792&454 | 32.8 |
| 5uja_B | -63.67 |  |  |  |  |  |  |  |  |
| 5w81_A | -41.29 | 335&1139 | 12.6 | 175&974 | 33 | 271&1069 | 21.5 | 572&1292 | 15.5 |
| 5w81_B | -45 |  |  |  |  |  |  |  |  |
| 6bhu_A | -25.63 | 580&1230 | 18.6 | 417&1065 | 31.5 | 512&1060 | 19.5 | 792&1454 | 17.8 |
| 6bhu_B | -23.02 |  |  |  |  |  |  |  |  |
| 6bpl | -84.93 | 281 | 7.8 | 118 | 29.9 | 213 | 28.7 | 506 | 20.2 |
| 6c0v_A | -10.35 | 329&972 | 14.2 | 165&802 | 33.1 | 261&904 | 20.9 | 556&1201 | 14.4 |
| 6c0v_B | -19.67 |  |  |  |  |  |  |  |  |
| 6fn4_A | -85.55 | 327&970 | 14.1 | 165&806 | 31.1 | 259&903 | 26.6 | 555&1200 | 19.7 |
| 6fn4_B | -72.26 |  |  |  |  |  |  |  |  |
| 6hrc | 0.8 | 290 | 16.6 | 126 | 30.7 | 221 | 17.8 | 510 | 13.2 |
| 6msm | -36.8 | 334&1131 | 13.5 | 174&966 | 32 | 268&1061 | 16.4 | 573&1271 | 15.5 |
| 6qee_A | -87.78 | 327&970 | 13.6 | 164&805 | 38.1 | 260&903 | 34.1 | 555&1200 | 26.1 |
| 6qee_B | -84.19 |  |  |  |  |  |  |  |  |
| 6quz_A | -4.53 | 270&294 | 20 | 106&130 | 30 | 201&225 | 15.4 | 495&517 | 13.8 |
| 6quz_B | 2.57 |  |  |  |  |  |  |  |  |
| 6qv2_A | -3.93 | 270&294 | 20.1 | 106&130 | 31.2 | 201&225 | 15.4 | 495&517 | 13.6 |
| 6qv2_B | 1.11 |  |  |  |  |  |  |  |  |
| 6raf_A | -86.61 | 275&290 | 9.7 | 112&127 | 21.1 | 208&223 | 30.4 | 500&523 | 27.3 |
| 6raf_B | -64.64 |  |  |  |  |  |  |  |  |
| 6rag_A | -107.09 | 275&290 | 9.9 | 112&127 | 29.7 | 208&223 | 32.8 | 500&523 | 25.4 |
| 6rag_B | -94.21 |  |  |  |  |  |  |  |  |
| 6rah_A | -9.19 | 275&290 | 26.9 | 112&127 | 29.7 | 208&223 | 18.5 | 500&523 | 14.6 |
| 6rah_B | -12.44 |  |  |  |  |  |  |  |  |
| 6rai_A | -15.64 | 275&290 | 10.4 | 112&127 | 29.4 | 208&223 | 21.6 | 500&523 | 14.8 |
| 6rai_B | -27.8 |  |  |  |  |  |  |  |  |
| 6ral_A | -26.39 | 275&290 | 9.9 | 112&127 | 29.5 | 208&223 | 19.6 | 500&523 | 15.3 |
| 6ral_B | -35.32 |  |  |  |  |  |  |  |  |
| 6pam_A | -57.51 | 298 | 10.8 | 134 | 28.7 | 231 | 30.9 | 523 | 18.8 |
| 6pam_B | -73.71 | 298 | 11.5 | 134 | 31.5 | 231 | 28.5 | 523 | 18.6 |
| 6pan | -15.17 | 298 | 14.7 | 134 | 25.2 | 231 | 21.9 | 523 | 13.4 |
| 6pao | -17.01 | 298 | 25.3 | 134 | 25.7 | 231 | 21.6 | 523 | 12.9 |
| 6paq | -15.42 | 298 | 18.4 | 134 | 25.2 | 231 | 21.4 | 523 | 13.6 |
| 6par | -29.28 | 298 | 10.3 | 134 | 25.4 | 231 | 21.3 | 523 | 13.5 |
| 6vqt | -131.4 | 298 | 10.5 | 134 | 46.6 | 231 | 45.9 | 523 | 44.7 |
| 6vqr | -23.27 | 298 | 11.6 | 134 | 26.2 | 231 | 22.6 | 523 | 15.4 |
| 7n58 | -128.9 | 417 | 11.2 | 252 | 42.2 | 349 | 44.9 | 641 | 45 |
| 7n59 | -100.4 | 417 | 10.6 | 252 | 38.8 | 349 | 38.9 | 641 | 36.3 |
| 7n5a | -25.82 | 417 | 12.4 | 252 | 26.4 | 349 | 22 | 641 | 14.8 |
| 7n5b | -15.71 | 417 | 19.1 | 252 | 25.3 | 349 | 21.4 | 641 | 14.1 |
